# Modular Robotic Limbs for Astronaut Activities Assistance

**DOI:** 10.3390/s21186305

**Published:** 2021-09-21

**Authors:** Sikai Zhao, Jie Zhao, Dongbao Sui, Tianshuo Wang, Tianjiao Zheng, Chuanwu Zhao, Yanhe Zhu

**Affiliations:** 1 State Key Laboratory of Robotics and Systems, Harbin Institute of Technology, Harbin 150001, China; 16b908056@stu.hit.edu.cn (S.Z.); jzhao@hit.edu.cn (J.Z.); suidongbao@hit.edu.cn (D.S.); 1110810113@hit.edu.cn (T.W.); zhengtj@hit.edu.cn (T.Z.); 2 Institute of Systems Engineering, China Academy of Engineering Physics, Mianyang 621900, China; skye908056@gmail.com

**Keywords:** astronaut operation, extravehicular activities assistance, robotic limbs, modular robots, wearable robots, reinforcement learning

## Abstract

In order to meet the assist requirements of extravehicular activity (EVA) for astronauts, such as moving outside the international space station (ISS) or performing on-orbit tasks by a single astronaut, this paper proposes an astronaut robotic limbs system (AstroLimbs) for extravehicular activities assistance. This system has two robotic limbs that can be fixed on the backpack of the astronaut. Each limb is composed of several basic module units with identical structure and function, which makes it modularized and reconfigurable. The robotic limbs can work as extra arms of the astronaut to assist them outside the space station cabin. In this paper, the robotic limbs are designed and developed. The reinforcement learning method is introduced to achieve autonomous motion planning capacity for the robot, which makes the robot intelligent enough to assist the astronaut in unstructured environment. In the meantime, the movement of the robot is also planned to make it move smoothly. The structure scene of the ISS for extravehicular activities is modeled in a simulation environment, which verified the effectiveness of the proposed method.

## 1. Introduction

In recent years, space exploration has been regarded as an important strategic development direction by the aerospace powers and relevant independent research institutions in the world [1]. With the rapid development and application of robot technology and artificial intelligence, the improvement of relevant technologies in the space exploration field have also been promoted significantly [2,3,4,5]. Although some space tasks have been replaced by robots gradually [6], the complex and smart operation tasks are still hard for robots to take over. Human operating ability and rich experience still play irreplaceable roles in executing high skilled tasks. Therefore, manned space exploration is still an effective means of space exploration and on-orbit service.

For the EVA on orbit service, astronauts usually need to complete long-time and complex tasks, such as the on-orbit assembly, maintenance and so on. When they move outside the cabin, they usually have to climb and move only under the assistance of safety rope, which is consumes a lot of energy and is apt to cause fatigue and trauma for the astronauts [7]. In addition, according to the report, there will be rough and sharp protrusions on the holding railings of the space station due to the high-speed impact of space particles [8], which usually tear the gloves worn by the astronauts during climbing. The above problems will affect astronauts’ safety and greatly limit the working duration of extravehicular activities.

To alleviate the above-mentioned problems, the space robots for assisting astronauts have been developed. The space manipulator can be used for on-orbit assembly, ISS maintenance, and long-distance motion assistance for astronauts outside the cabin, such as the Space Station Remote Manipulator System (SSRMS) [9] and the Special Purpose Dexterous Manipulator (SPDM) [10]. The anthropomorphic astronaut robots are mainly used for demonstration and verification tests of shaking hands with astronauts or delivering tools, such as the Robonaut 2 (R2) [11,12], and Skybot f-850 [13]. Researchers have also proposed the space robots which can adapt themselves to complex and changeable space environment and also have the capability of on-orbit fabrication or assembly [14,15]. In the meantime, in order to improve the robots’ adaptability to the changes of its own state and environment, researchers have introduced intelligent algorithms to enable the robots’ self-modeling and self-evolution [16,17,18,19]. For the astronauts, these space robots only aim at providing assistance on wide-range movement outside the cabin, executing simple tasks in the cabin or carrying out self-assembly work at the conceptual level, but they are constrained in small-range movement or complex operation outside the cabin. Besides, NASA has developed an astronaut assisted exoskeleton robot system (X1) to enhance the physical ability of astronauts when walking out of the cabin in the future [20]. However, these assisted devices may not only constrain the mobility of astronauts’ body, but also interfere with their spacesuit.

The wearable robotic limbs can provide a novel aid in the face of such difficulties. They can serve as the extra arms of human and provide help during the operation. Asada et al. developed several wearable robotic limbs for Boeing aircraft manufacturing and the nuclear industry. Combined with the pilot’s limb posture and working process, they can realize moving assistance, working assistance in special position, human body support and so on [21,22,23,24]. Vatsal et al. [25] designed a extensible robotic limb worn on the forearm, to grasp objects. Gopinath et al. [26] carried out preliminary experimental tests to assist drummers to play drums using wearable robotic limbs. The waist wearable robotic limbs proposed by Sasaki et al. has two mechanical arms [27,28] and operated by human foot and toes. These wearable robotic limbs just aim at the needs of industrial manufacturing and daily assistance on the ground. They have not been involved in manned space exploration for astronaut operation assistance.

In this paper, the robotic limbs for astronauts are named AstroLimbs, and can help astronauts perform extravehicular activities. To our knowledge, it is the first time the concept of applying robotic limbs for astronaut assistance in the space exploration field has been proposed. The AstroLimbs are developed to offer climbing assistance for astronauts outside the cabin, by actively carrying astronauts to help them move together. Each limb is composed of six basic module units with identical structure and function. The basic unit has a rotational degree of freedom (DOF) and the modules are interchangeable. Based on the reinforcement learning method, the robot can move autonomously to the final target from any starting position. The simulation environment referring to the structure of the space station is constructed. The basic movements of the AstroLimbs are planned, including the joint angular velocity and acceleration, the variation of the robot’s posture and the appropriate distance between robot and its current work plane. Via these plans, the robot can move smoothly and stably. The rationality of the learning method and the moving performance are verified in the simulation environment. Thanks to this learned ability, the robot can free both hands of the astronauts to carry out extravehicular activities. In addition, it will also reduce the damage risk to the spacesuit and extend EVA duration.

The paper is organized as follows. Section 2 describes the utilization concept and the development of the wearable robotic limbs. Section 3 mainly introduces the method of learning to move autonomously and plans the movement of the robotic limbs. Section 4 mainly presents the verification of the learning method and the related results in the simulation environment. Finally, Section 5 summarizes the work of this paper and looks forward to future work.

## 2. The AstroLimbs Design

### 2.1. The AstroLimbs Concept

**Figure 1:**
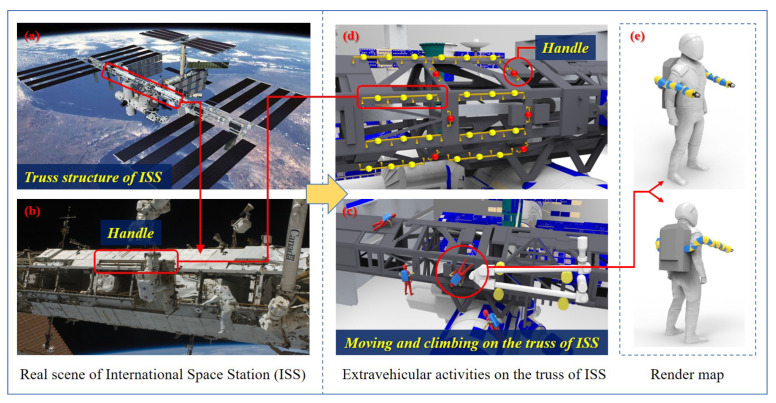
Simplified environment of ISS, and the render map of the AstroLimbs. (**a**,**b**) Real scene of ISS. (**c**,**d**) Extravehicular activities on the truss of ISS. (**e**) Render map of the AstroLimbs.

At present, human manned space activities mainly rely on the space station, which is equivalent to a space laboratory with modern scientific research equipments and is capable of conducting scientific experiments in microgravity environment. It also provides a platform for astronauts to stay in earth orbit for a long time in space, and makes it convenient for astronauts to carry out space experiments, extravehicular walking and other on-orbit activities. In the meanwhile, astronauts need to carry out the extravehicular assembly, inspection and maintenance of the space station. Figure 1 shows the real and simulated scenes of the ISS, and the simulation scene of astronauts’ extravehicular activities assisted by wearable robotic limbs. The structural design of the ISS is based on the truss structure, which constitutes the keel frame of the space station, as shown in Figure 1a,b. Other multi-function modules, extravehicular service equipment, large mechanical manipulator service systems and solar panels are all installed on the keel frame, which forms a truss hanging cabin type space station. On one hand, it ensures the overall stiffness of the space station, and is conducive to the normal independent operation of each subsystem. On the other hand, it can provide a foothold for astronauts’ assembly and maintenance activities. As shown in Figure 1c,d, astronauts rely on the truss of the space station to move, climb and work outside the cabin. The red frame marks represents the handles installed on the truss for facilitating climbing and moving.

Based on the scale relationship between the truss of ISS and the astronauts in Figure 1a,b, the corresponding simulation environment is shown in Figure 1c,d. Figure 1c shows the scene of astronauts moving, climbing, and working on the truss of ISS with the assistance of the AstroLimbs. In the simulation modeling, the cross section of the truss of ISS is designed as regular hexagon shape, which is an axisymmetric figure. Thus, this research mainly focuses on the three work planes that can be seen in the front view. As shown in Figure 1d, the handles for astronauts moving is designed according to the real counterparts in Figure 1b and can be classified into horizontal one and vertical ones in terms of the installation method. Its mounting position refers to the relative position of the real handle on the truss structure in Figure 1b. Two rows of horizontally installed handles are designed on the front work plane. The other two work planes intersecting with the front work plane have one row of horizontally installed handles, respectively. The truss of ISS is equidistantly divided into several space areas, and the corresponding climbing handles are vertical installed handles and are placed on the partition wall in the middle of each area, as shown in the red circle in Figure 1d. These two types of handles provide connection places for astronauts to move freely in a small range on the truss structure outside the ISS.

### 2.2. Mechanical Design

Figure 1e shows the concept of AstroLimbs for assisting extravehicular activities. The AstroLimbs has two robotic arms, which are fixed near the back waist of the astronaut and connected to the astronaut’s backpack. The AstroLimbs grants the pilot two more arms, which can assist the astronaut to climb on the truss outside the cabin and improve the operation ability of addedexecuting extravehicular activities. Besides, when in the fixed working position, it can serve as a partner for the astronaut to carry out the tasks which are difficult to complete for a single person.

**Figure 2:**
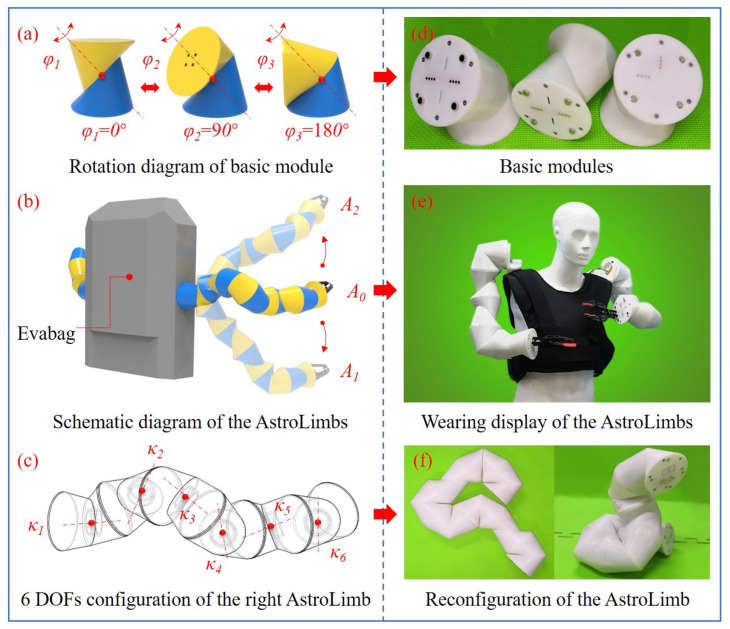
Wearable robotic limbs for astronaut extravehicular activities assistance. (**a**) Rotation diagram of basic module. (**b**) Schematic diagram of the AstroLimbs. (**c**) 6 DOFs configuration of the right AstroLimb. (**d**) Basic modules. (**e**) Wearing display of the AstroLimbs. (**f**) Reconfiguration of the AstroLimb.

The AstroLimbs system is designed to be modularized due to the following advantages. The basic module unit will have the same structure and function, which can be quickly assembled and disassembled. It is able to improve the fault tolerance of the AstroLimbs when working in the outer space. If one basic module unit of the AstroLimbs has a problem, it can be replaced by another intact module. The basic module units, the reconfiguration and the wearing display of the AstroLimbs are shown in Figure 2. Each basic module has an independent rotational DOF, as shown in Figure 2a,d. Figure 2a is the 3D render map of the basic module unit. Each basic module unit is composed of two identical sub-modules that connected by a steering engine.

Each sub-module is made of a deformed triangular prism with the edge ground to be smooth surface, which is distinguished by the yellow and blue entities in Figure 2a. At the same time, each sub module has a rotation plane and a connection plane. The rotation planes of the sub-modules coincide with each other to form the rotational DOF. The common axis of rotation is perpendicular to two rotation planes. Its rotation range is −180–180${}^{\circ }$. Figure 2a shows the rotation of the module at 0${}^{\circ }$, 90${}^{\circ }$ and 180${}^{\circ }$, respectively. The situation will be similar when rotating in the other direction. The prototypes corresponding to the rendering basic modules are shown in Figure 2d. In the meanwhile, the AstroLimbs contains a complete electrical system to move the arm. Metal contacts for electrical connection are designed for connecting adjacent modules. They are used for power supply and communication between modules, to ensure the fast and reliable connection of the electrical system.

Combined with the idea of anthropomorphic bionic design, two extra arms are designed in the robot system according to the needs of auxiliary operations. The number of modules in each arm can be arbitrarily configured. The total freedom of the robotic arm is determined by the number of modules connected in series. In order to ensure the basic spatial movement ability, the robotic arm was designed with 6 DOFs. Namely, each arm is equipped with six basic modules as the target model for using in the space exploration, as shown in Figure 2b. Figure 2e just shows the wearing display of the AstroLimbs. Evabag shown in Figure 2b is the EVA backpack for outer spacewalk of astronauts. Two wearable robotic limbs are fixed on both sides of the Evabag and are located near the place between waist and chest of the astronaut. This mounting position is good for the robotic limbs to assist the astronaut to move, operate with other astronauts and reduce the spatial overlap with the human limbs. In Figure 2b, *A${}_{0}$*, *A${}_{1}$* and *A${}_{2}$* represent the status of the AstroLimbs’ right arm at three different positions, respectively. The configuration of rotating DOFs corresponding to the right robotic limb in Figure 2b is shown in Figure 2c, where κ${}_{1}$–κ${}_{6}$ represents the six different rotating shafts of active DOFs. The module with rotating shaft κ${}_{1}$ is fixed to the Evabag. The module with rotating shaft κ${}_{6}$ is the operating end of the robotic limb. When the rotation angle of each joint is given, the spatial position and posture of the end point relative to the selected base coordinate can be obtained through the forward kinematics of the robotic limb. The state *A${}_{0}$* can be set as the initial state, when the six joint angles varies, the position and posture of the end actuator will also change accordingly. For example, it can move to a new position as illustrated by state *A${}_{1}$* or state *A${}_{2}$* in Figure 2b. Figure 2f shows the reconfiguration of the real robotic limb with multiple DOFs based on the modules connected in series.

## 3. Method for Autonomous Motion

### 3.1. Q-Learning Algorithm

Q-learning algorithm of reinforcement learning is introduced for achieving the autonomous motion ability of the AstroLimbs with target orientation at any point on the truss of the ISS during climbing. Therefore, based on the designed working environment of the truss, the state of the AstroLimbs system is divided, and the basic motion of the AstroLimbs is planned. The reward function in the process of task learning is proposed. The corresponding analysis of the training and evaluation results based on reinforcement learning is conducted.

Reinforcement learning is an overall process refers to the agent’s trial, evaluation and memory. The agent’s learning maps from environment state to action, which makes the agent gain the maximal reward after executing a certain action. This learning process will make the agent perform best under some preset evaluation rules. Q-learning algorithm is one of the evaluation rules for the agent to choose a specific action in the current state, which is an action-utility function. Q is short for the word of quality, which is the quality feedback to each action and provides memory for the agent. When the number of states and actions in the learning process is limited, Q-learning algorithm is very suitable for model free autonomous motion planning.

Each time in a specific state, the corresponding evaluation value of the agent after executing the action has the following expression:

(1) $$Val=max_{a}Q\left(s,a\right)$$

where, *s* is the current state, *a* is the action that can be taken in the current state, and *Val* is the obtained maximal value of the evaluation corresponding to this action under the conditions of the current state *s* and action *a*, based on this value the agent can determine the action to execute in this step.

The core of Q-learning algorithm is the process of constantly updating the evaluation value *Val* in Equation (1) according to the continuous trial training:

(2) $$newQ\left(s,a\right)⇐Q\left(s,a\right)+\lambda \left[R\left(s,a\right)+\eta \cdot max_{a^{\prime }}Q\left(s^{\prime },a^{\prime }\right)-Q\left(s,a\right)\right]$$

where *R* represents the reward value that can be obtained by executing action *a* in the current state *s*, *s*’ is the new state of agent after executing action *a*, *a*’ is the possible action in state *s*’, λ is the learning efficiency (λ = 0.01) and η serves as the discount factor (η = 0.9).

### 3.2. Determination of the States

**Figure 3:**
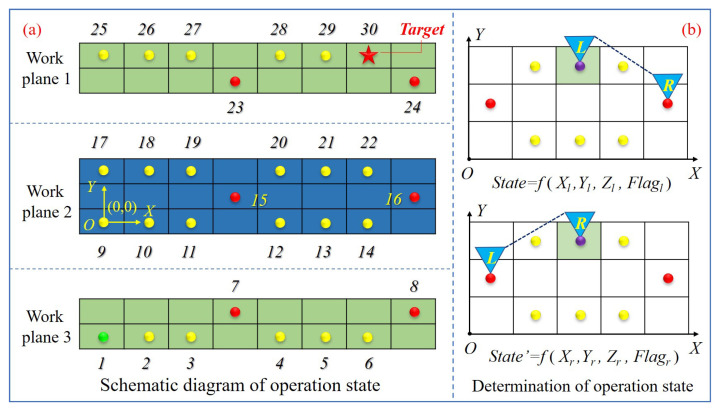
Determination of the AstroLimbs state. (**a**) Schematic diagram of operation state. (**b**) Determination of operation state.

Figure 3a shows the schematic diagram of the operation status on the ISS truss where the yellow dot, red dot, green dot and red pentagram refer to the handles that can be grasped by the AstroLimbs during the movement. There is a total of 30 handles and each handle represents the corresponding status in Figure 3a. Among the handles, the green dot represents the starting point of the training process (Point 1). The red pentagram stands for the target point (Point 30). According to Figure 3b, as the AstroLimbs has two robotic arms, the same foothold (the foothold on the grid colored with green) may belong to either the end of the left arm or the right arm. At this time, although the foothold position is the same, yet it must be regarded as two different states. Thus, there are total 60 states in the process of moving, and the state value can be judged with the following expression:

(3) $$State=f(X_{k},Y_{k},Z_{k},Flag_{k})$$

where, *State* is the state number of the AstroLimbs moving on the truss of the ISS, *k* is the current moving arm of the AstroLimbs, *Flag${}_{k}$* is the corresponding identification of the current moving arm, when *k* = left, *Flag${}_{k}$* = 1 (*k* = right, *Flag${}_{k}$* = 0), (*X${}_{k}$*, *Y${}_{k}$*, *Z${}_{k}$*) are the landing point coordinates of the AstroLimbs’ moving end.

### 3.3. Setting of the Actions

The moving actions of the AstroLimbs will be affected by its own structure and environmental factors. The main influencing factors are listed as follows: (1) The structural features and size of the AstroLimbs itself; (2) The moving ability of the AstroLimbs; (3) The work plane of the state point (the foothold); (4) The position relationship between the state points. Based on the above constraints, the effective actions of the AstroLimbs are introduced, assuming that the left end of the AstroLimbs is fixed, and only the right end are free. Finally, 14 kinds of effective moving actions are obtained and the relative coordinates of the two ends are processed equivalently.

The equivalent coordinate is introduced to show the relative position of the robot left and right ends. For the X and Y directions, the meaning of equivalent coordinate values (*x${}_{eq}$* and *y${}_{eq}$* ) represent the number of handle intervals (or the number of unit intervals) in the right end relative to the left one in the corresponding coordinate direction. In any direction, the distance between the two nearing points is regarded as one basic unit. At this time, if the right end is two units away from the left one, the corresponding equivalent coordinate will be two. When the right end below the left one, the value will turn to be −2. In addition, due to above mentioned influencing factors for the robot motion, the maximum movement distance of the robotic limb in each direction is two basic units. Therefore, the lower and upper limit of the equivalent coordinate values (*x${}_{eq}$* and *y${}_{eq}$*) are −2 and 2, respectively.

However, for the Z direction, the equivalent coordinate value *z${}_{eq}$* is designed to distinguish between various situations when the two ends are in different work planes. It determines the spatial moving action mode of the AstroLimbs and the value is self-designed to be from −2 to 2 (*z${}_{eq}$*∈{0, 1, −1, 2, −2}). As shown in Table 1, action mode *A* means that the AstroLimbs moves in the same work plane and the value *z${}_{eq}$* is set at 0. Action mode *B* means that the right end is in the work plane 1 and the left end is in the work plane 2 (*z${}_{eq}$* = −1). Action mode *C* means that the right end is in the work plane 2, the left end is in the work plane 1 (*z${}_{eq}$* = 1). Action mode *D* means that the right end is in the work plane 3 and the left end is in the work plane 2 (*z${}_{eq}$* = −2). The action mode *E* means that the right end is in the work plane 2, the left end is in the work plane 3 (*z${}_{eq}$* = 2).

According to Table 1, {*x${}_{eq}$, y${}_{eq}$, z${}_{eq}$*} is the equivalent coordinate (Eq-Coordinate) of the right end relative to the left end, as shown in Figure 3b. *x${}_{eq}$* represents the interval number of the state points of the right end relative to the left end in the *X*-axis direction, and the value of *x${}_{eq}$* can be obtained (*x${}_{eq}$*∈{1, 2}). In the same way, *y${}_{eq}$* represents the interval number of the state points in the *Y*-axis direction and then *y${}_{eq}$*∈{0, 1, −1} can be obtained, where the sign represents the front and back position relationship between the two ends. Especially, the minus sign represents that the left end is in front of the right side in terms of the *Y*-axis. For example, the equivalent coordinates of the two actions are {2, −1, 0} and {2, 1, 0}, respectively, in Figure 3b.

Similarly, when the right end is fixed, and only the left end are free, the equivalent coordinates are the same if the relative position relationship between the two ends remains unchanged. Thus, a total of 28 effective actions for the AstroLimbs can be expressed in the form of 14 equivalent coordinates, as shown in Table 1.

### 3.4. The Construction of Reward Mechanism

Reward is the expression of the contribution made by the agent to perform a specific action in a specific state when achieving the task goal. The goal of the agent is to maximize the expected cumulative reward. During the training process, there are three basic cases for the AstroLimbs in this paper. The first one is the success case, when the robot gets to the final target. The second one is the common case, in which the robot reaches the designed state points except the target. The last is the failure one, when the robot moves out of the allowable points. When the robot steps to another case, the corresponding reward will also turn to be different. Once the robot reaches the target, it will be given the maximal reward. The construction of reward mechanism for the robot will directly affect training process and result. In order to ensure the AstroLimbs has an equal chance to be trained at each state point except the final target, all the rewards of the state points will be set to be the same and the value is designed to be zero. In addition, if the AstroLimbs moves out of the boundary of the working area or its moved end place does not belong to any state that has been planned, it is regarded as failure of the task. In this case, the agent will be punished and the reward is set to a negative value. Based on those principles, we can establish a reward mechanism, which can be expressed by the following Equation:

(4) $$R=\left\{\begin{array}{cc} R_{t}, & U_{e}\in \left\{S_{k}|k=30\right\}\\ 0, & U_{e}\in \left\{S_{k}|k=1∼29\right\}\\ -R_{t}, & U_{e}\notin \left\{S_{k}|k=1∼30\right\} \end{array}\right.$$

where, *R* is the reward value obtained by the agent after performing a specific action, *R${}_{t}$* is the maximal reward value when the robot reaching the target and the value is designed to be 50, *U${}_{e}$* is the current state of the robot after executing the action, and {*S${}_{k}$*} is a set of all the effective states in the process of the robot moving which is planned in advance as shown in Figure 3a.

For the training process of the AstroLimbs, when stepping into the common state1–state29, the value *R* for the robot will always be 0. It means the robot has neither reward nor punishment. The robot will be greatly encouraged by acquiring the maximum reward, the value of which *R* is 50, when reaching the final target state30. Apart from the allowable states, if the robot gets into an undesirable state, it will be punished by deducting a certain reward value of 50 and the value *R* is −50.

### 3.5. Movement Planning of the AstroLimbs

**Figure 4:**
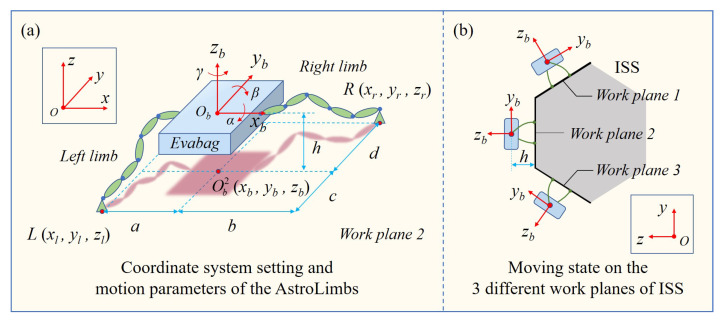
Simulation environment setup based in the Vrep. (**a**) Coordinate system setting and motion parameters of the AstroLimbs. (**b**) Moving state on the three different work planes of ISS.

In order to plan the basic movement of the AstroLimbs, it is necessary to simplify the overall modeling of the astronaut-robot system, and set the basic assumptions and constraints based on the operation requirements. Due to the astronauts and the backpack are integrated, the astronaut and the space backpack can be regarded as a whole, which will be represented by Evabag (the blue box) as shown in Figure 4.

The AstroLimbs has two robotic limbs with six DOFs, represented by the green ellipse module units. The mechanically connected parts of the two basic module units in the series arm are regarded as a solid whole, represented by the green ellipse in Figure 4a. The blue circle between the two green ellipses in Figure 4a represents a rotational DOF. Due to the direction design of the rotational DOFs of the robotic limbs, these rotation shafts are not parallel to each other to guarantee the position and posture of the AstroLimbs’ end effector in the working space. Thus, the AstroLimbs system has 12 DOFs as a whole. The coordinate system *XYZ* serves as the world coordinate system {0} and is also the coordinate system of the ISS truss. The coordinate system {B} is fixed on the Evabag, whose origin *O${}_{b}$* coincides with the mass center of the Evabag. The plane *Y${}_{b}$O${}_{b}$Z${}_{b}$* is set as the sagittal plane of the astronaut, in which the axis *y${}_{b}$* points to the head direction of the astronaut and the axis *z${}_{b}$* points to the back of the astronaut. Therefore, based on the movement of the astronaut, the α, β and γ refer to pitch angle, roll angle and yaw angle of the astronaut movement, respectively.

Due to the fact that the simplified system has 12 DOFs, there are still six redundant DOFs when the position and direction of the two arms’ ends are determined. In order to better plan the basic moving action of the AstroLimbs and meet the astronauts’ comfortable and working needs on the truss of the ISS, it is necessary to set the basic restrictions on the movement position and posture of the astronaut-robot system during moving. These basic restrictions can be described as following:

(1) The moving position of the Evabag: The projection point of the mass center *O${}_{b}$* of Evabag on the corresponding work plane should be within a certain range near the middle point coordinate position of the double ends of the two robotic limbs. The ideal position is designed as:

(5) $$\left|O_{b}^{i}O_{m}^{i}\right|<\delta$$

where, *i* represents the corresponding work plane (*i* ∈ {1,2,3}), *O*${}_{b}^{i}$ is the projection point of Evabag’s mass center in the work plane *i*, *O*${}_{m}^{i}$ is the middle point coordinate position of the AstroLimbs’ two end points, δ is the allowable deviation from the center position, which is related to the values of *a*, *b*, *c* and *d* in Figure 4a.

(2) Space for the pilot to work: A certain distance from the mass center of Evabag to the work plane should be ensured to leave space for the astronaut to move and work without restriction and interference, as shown in the Figure 4b. This distance should be neither too large nor small. On the one hand, it might be limited by the length of the robotic limb, but on the other, a close enough distance can increase the safety factor for the astronaut to deal with emergencies. Therefore, the space constraint for the astronaut’s movement can be expressed as:

(6) $$h_{d}\leq \left|O_{b}O_{b}^{i}\right|\leq h_{u}$$

where, *h*${}_{d}$ and *h*${}_{u}$ stand for the lower and upper limits of the space reserved for the astronaut to work, respectively.

(3) Stability of the movement: To ensure that the astronauts will not suffer from excessive pitch, roll and yaw in the process of moving, the Evabag’s movement direction can be limited as follows:

(7) $$\left(\left|\alpha \right|\leq \theta_{\alpha }\right)\cap \left(\left|\beta \right|\leq \theta_{\beta }\right)\cap \left(\left|\gamma \right|\leq \theta_{\gamma }\right)$$

where, α, β and γ refer to the pitch angle, roll angle and yaw angle of the astronaut respectively as shown in Figure 4a, θ${}_{\alpha }$, θ${}_{\beta }$ and θ${}_{\gamma }$ are the corresponding restrictions of the angles that mentioned above. These three limitations will determine the attitude of the astronaut in the process of extravehicular activities.

(4) Movement limitation: in the meanwhile, for each limb of the robot is composed of six basic modules connected in series, whose envelope surface has a cylindrical shape with a height of 0.1 m. So the length of each limb is 0.6 m. According to the setting of the simulation platform and the dimensions of the robot system, the AstroLimbs’ ability of moving cross the landing handles is determined. It also can be expressed by the following Equation:

(8) $$\left(\left|\vec{P_{x}}\right|\leq \rho_{x}N_{x}\right)\cap \left(\left|\vec{P_{y}}\right|\leq \rho_{y}N_{y}\right)\cap \left(\left|\vec{P_{z}}\right|\leq \rho_{z}N_{z}\right)$$

where, $\vec{P}$ is the direction vector from the left arm end point to the right arm end point, $\vec{P_{x}}$, $\vec{P_{y}}$ and $\vec{P_{z}}$ are the projection of the vector on the *x*, *y* and *z* axes, respectively, $\rho_{x}$, $\rho_{y}$ and $\rho_{z}$ are the basic preset moving span in the three-axis directions, *N*${}_{x}$, *N*${}_{y}$ and *N*${}_{z}$ are the maximal interval value of the footholds (*N*${}_{x}$ = 12, *N*${}_{y}$ = 5 and *N*${}_{z}$ = 4.5).

Based on Vrep simulation platform, combined with the motion constraints of the robotic joints, the basic moving actions for the AstroLimbs can be planned according to the Equations (1)–(8). In the meantime, through the inverse kinematics solution, the movement angles of each joint corresponding to the basic moving actions can be obtained. In order to give the AstroLimbs the capability of moving smoothly, the moving position, velocity and acceleration of the joints are planned. Considering that there are two kinds of motion modes in the process of moving on the truss, one is the continuous motion between each step, and the other will stop at a certain foothold for some time and then perform the next step. As the requirement for the former mode is more demanding, trajectory planning for the continuous movement will be considered. In order to ensure the continuity of each step and the stability of the moving action switching, the velocity *V*${}_{ei}$, acceleration *A*${}_{ei}$ of the AstroLimbs’ moving end on each landing point are set to zero. The maximal velocity *V*${}_{max}$ (90${}^{\circ }$/s) and maximal acceleration *A*${}_{max}$ (20${}^{\circ }$/s${}^{2}$) are also limited.

## 4. Simulation and Results

### 4.1. Construction of Simulation Environment

**Figure 5:**
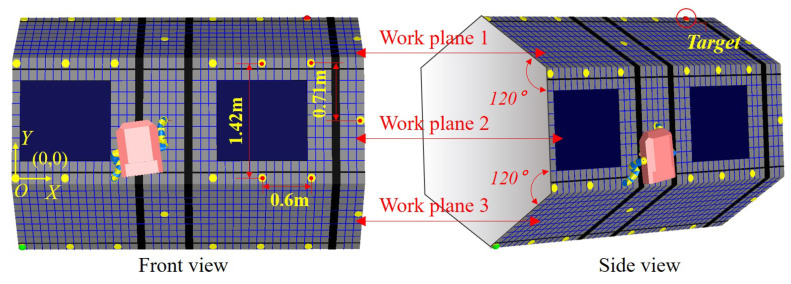
Simulation environment setup based on the Vrep.

According to the working environment designed in Figure 1c,d, the integrated platform involving the astronaut, the robotic limb and working environment is further simplified based on the Vrep simulation environment. Figure 5 shows the front view and side view of the simplified simulation platform. The three visible surfaces of the truss are work plane 1, work plane 2 and work plane 3. The work plane 2 is the front view plane, the work plane 1 and work plane 3 are distributed 120${}^{\circ }$ apart from work plane 2. The astronaut and the AstroLimbs can move, climb and work on the three planes. The dimension parameters are shown in the work plane 2, the span of two neighboring handles is 0.71 m in the *Y*-axis direction, and it is 0.6 m in the *X*-axis direction. The dimension parameters setting is also suitable for the work plane 1 and work plane 3. These distance parameters are estimated according to the proportional relationship between the ISS and astronauts, as shown in Figure 1b. This paper aims to verify the principle of the proposed method with the developed AstroLimbs, which will not be affected seriously by the actual size of the work scene. Relevant practical parameters will be confirmed facing future manned space application.

### 4.2. Training Results and Evaluation

**Figure 6:**
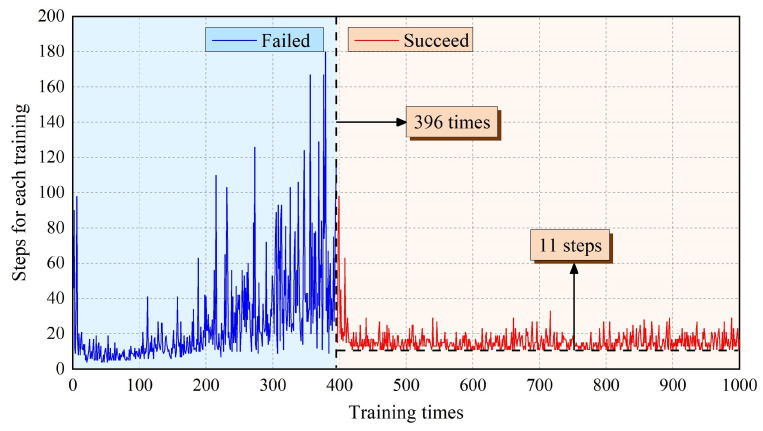
The number of steps of AstroLimbs for each training.

**Figure 7:**
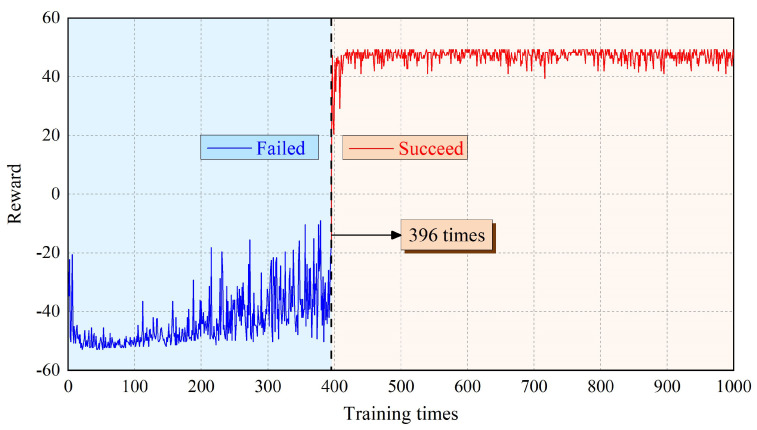
Reward value of AstroLimbs for each training.

For the training process, the robot is reset to be in the position of State1 (i.e., the starting point of the task). Thus, the end of left arm will be set in State1, and the end of right arm is in State2. Then the target point of this task is always fixed in State30. The robot will start from the initial point and move two robotic limbs alternately. Each movement will be recorded as one step. When the robot reaches the target point, it completes this training successfully. The number of training times is designed to be 1000. During the training process, the number of steps required to reach the target point should be recorded. In the meanwhile, the cumulative reward of each training episode will also be focused on. The training results can be seen in Figure 6 and Figure 7.

**Figure 8:**
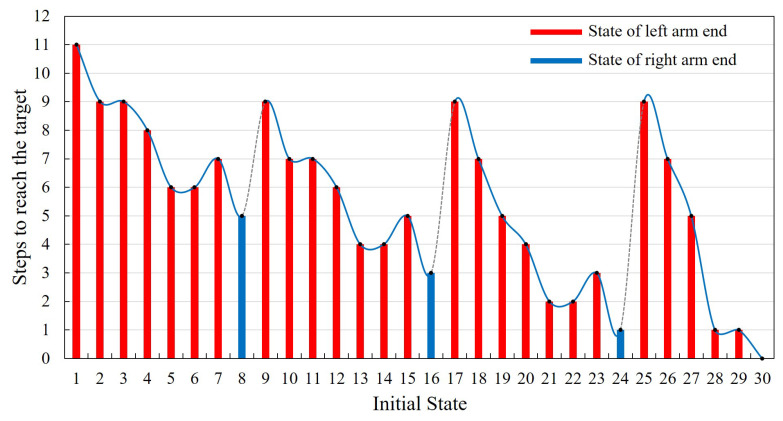
The number of steps required for the AstroLimbs to reach the target from different state points.

Figure 6 shows the steps that AstroLimbs needed to reach the final target. The horizontal ordinate stands for the training times and the vertical ordinate represents the step number required to reach the target point for each training episode. Once the robot moves out of bounds, it will be regarded as the failure of this training task and the robot will be reset to the starting point. In this situation, there is no definite number of steps to finish the moving task. That is to say, there are two possible results for the training. One is abnormal movement in which the AstroLimbs might go out of the boundary and fail to reach the target. The other is reaching the goal successfully. As shown in Figure 8, the blue area on the left represents training failure, and the red area on the right represents training success. During the first 1–395 training episodes, the AstroLimbs failed to get to the target. After 395 failures, the robot can reach the target point successfully on the 396th training and performed well after that.

At the beginning of the training process, the robot is still an inexperienced agent who knows nothing about the surrounding environment. Thus, it will need a certain amount of trial trainings to accumulate the basic experience, which is more likely to step out of the prescribed bounds and fail to reach the target position. In this stage, the steps to reach the target is defined to be the number of moving steps before the failure. It can be seen from the figure that the value is between 4–180, and the data trend is to increase slowly and then decrease. After a variety of failing trials, the number of steps required in the process of 396–430 training times is gradually decreasing. At this stage, the robot has accumulated a certain amount of experience by the earlier trails. Based on this experience, the robot has acquired the ability to understand the environment to some extent and has the chance to finish the task successfully. Thus, the steps needed to reach the target point will decrease.

However, it has not yet converged in this stage. After more than 430 times of training, the steps required for the robot decrease to the bottom and keep stable on the value 11. Due to the ε-greedy strategy, the robot still maintains a certain exploration ability in the later stage of the training process. Although the robot has obtained a route to the target point, it will still choose to explore new action sequence with a certain probability. Hence, there is still a small fluctuation of the convergence step number around the value 11, which has no effect on the step number convergence of the whole training process. It serves as the minimal step number the robot need to get the State30 from State1. The corresponding action sequence enables the robot to move autonomously towards to the finishing point.

Figure 7 shows the cumulative reward value obtained by each training episode for the robot. Corresponding to Figure 6, taking the 396th training as the boundary, it can also be divided into the blue area of failure and the red area of success. It can be seen from Figure 7 that for the first 430 training episodes, the cumulative reward obtained by the robot gradually increases from −50 to 50 ignoring small fluctuations, where the reward is consistent with the setting value. After 430 times of training, the cumulative reward fluctuates slightly, but still tends to be stable. The robot gradually understands the environmental information and acquires the ability to choose the appropriate action.

In addition, based on the training results, the autonomous movement ability should be evaluated as well. State30 is the final target point, and the initial positions are set to be from State1 to State30, successively. During this evaluation process, whether the robot can reach the final target point successfully will be recorded. If it can reach the target point, the steps of the robot’s autonomous movement will also be preserved, which can be introduced as the evaluation criterion of the learning result. The results are shown in Figure 8. In the process of autonomous moving ability evaluation, the initial position state of the robot is set as the position of the left arm end. It is similar for the right arm end. Thus, only the former condition will be taken as a representative for further analysis. Besides, only one limited section of the ISS truss structure is intercepted as the training environment, which can be sufficient to verify the proposed learning method. The positions of State8, State16 and State24 are the right boundary of the training environment. When the end of the robot is within the three positions, the robot will move out of the training boundary after taking the next action. Therefore, to carry out the evaluation normally as well, we set the position of the right arm end as its state. The movement results of these three special positions are marked by blue columns in the Figure 8. In addition, when in State30, the robot does not need to perform any action to achieve the goal, the number of steps is recorded to be zero.

Thus, the final results of the evaluation are shown in Figure 8. It is obvious that the robot succeeds in getting to the final target point from any position point. The corresponding number of steps required is 0–11, and the actual success rate is 100%. According to the evaluating results, as the distance between State1 and final target point is the farthest, it will take up to 11 steps to reach State30 from State1. The State24, State28 and State29 are closer to the final target point, thus only one step is needed. Comparing the state position and the relative step number the robot needed, there are some intrinsic relations between these two parts. According to the state definition in Figure 3, State1–State8 belong to work plane 3, State9–State16 belong to the lower part of work plane 2, State17–State22 belong to the upper part of work plane 2 and State23–State30 belong to work plane 1. From the variation trend of the required steps in the Figure 8, it can be found that for different parts of the work plane, the trend of the required steps from left to right varies similarly. Taking State1–State8 as an example, State1 has the largest number of steps. From State2 to State6 shows a declining trend gradually. Then State7 will increase and State8 turns to be the smallest. This trend of variation is related to the position of the state point in the x-axis direction. The closer to the final target place in the x-axis direction, the fewer steps are needed. Although the foothold in the middle of the work plane of the space station truss is closer to the target point than the state point with smaller number in the same plane, its step number will also increase due to the particularity of its position. The footholds in this kind of position, which have fewer close neighboring counterparts. These state points can be regarded as footholds with obstacle attribute, which will not be easy for the robot to pass, so it needs more steps.

According to the analysis of the training results and evaluation, through Q-learning algorithm, the robot gradually learns the state determination, optional position identification, environment boundary identification, task target confirmation and autonomous motion planning. Therefore, the target oriented autonomous motion ability on the ISS truss is obtained by the robot.

### 4.3. Simulation Result

Based on the mechanical design of the AstroLimbs, the construction of the simplified working structure and the establishment of the autonomous motion training mechanism, the training process for the AstroLimbs is carried out. In the meanwhile, the training process is also described in detail as follows. Due to the fact that the astronaut and backpack are connected together, the backpack (Evabag) will be used to replace the astronaut-backpack integrated system, which will have no effect on the final results and can obtain higher simulation efficiency.

**Figure 9:**
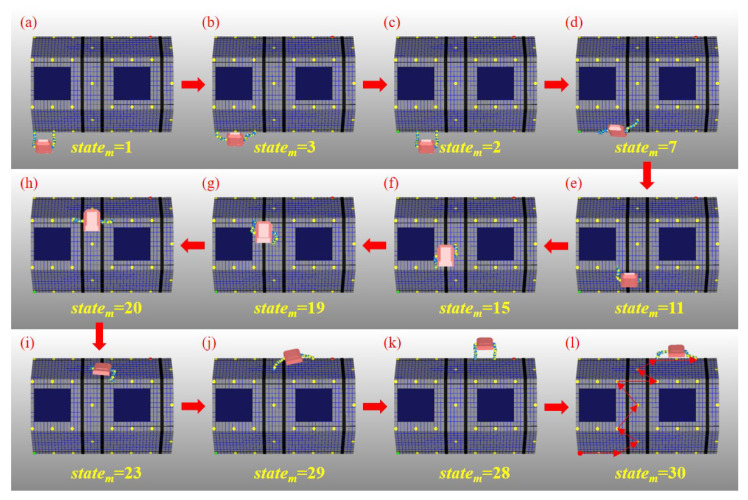
Sequential diagram of the autonomous movement simulation. (**a**) The state of the robot when standing at the initial point. (**b**) State after the 1st step. (**c**) State after the 2nd step. (**d**) State after the 3rd step. (**e**) State after the 4th step. (**f**) State after the 5th step.(**g**) State after the 6th step. (**h**) State after the 7th step. (**i**) State after the 8th step. (**j**) State after the 9th step. (**k**) State after the 10th step. (**l**) State after the 11th step to reach the final target.

All the moving actions that AstroLimbs has taken during the training process derives from the planning based on the Equations (5)–(8). The autonomous movement process of the AstroLimbs from the State1 to the target is shown in the Figure 9.

Through 1000 iterations of simulation training, the AstroLimbs system can obtain the ability of autonomous movement under the task goal orientation. The training result is shown in Figure 9a–l, which is the decomposition diagram of the autonomous movement of AstroLimbs from the initial place after the simulation training. Taking Figure 9b as an example, it represents that the robot takes the first action from the initial position and its current state (*state*${}_{m}$) turns to be State2. Similarly, Figure 9c–l correspond to the state of the AstroLimbs after taking each action. For example, due to Figure 9c, the left arm end is the last moved end, which has step into the point of State2. The AstroLimbs moves its double arms by turns and reaches the final target within 11 steps from the starting point. In the meanwhile, by connecting the state points that the robot has passed, we can obtain the route of autonomous movement, as shown in Figure 9l. This route colored by red is chose by the AstroLimbs itself after the training. According to Figure 9l, the robot moves along the direction of the connecting line between the starting point and the final target, and the route formed by the selected action sequence is short and reasonable.

**Figure 10:**
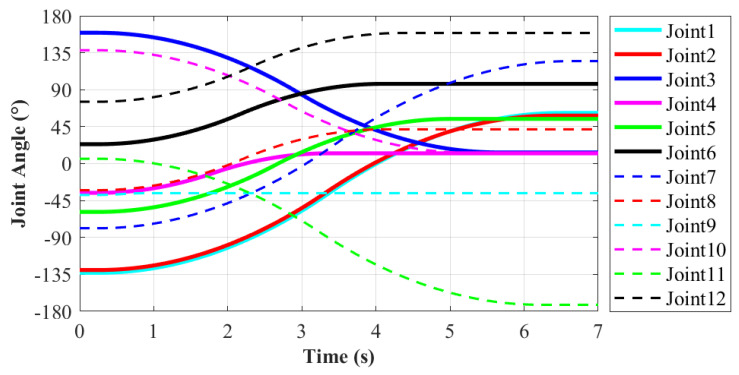
The angle variation of each joint. (Action 9 according to Table 1).

**Figure 11:**
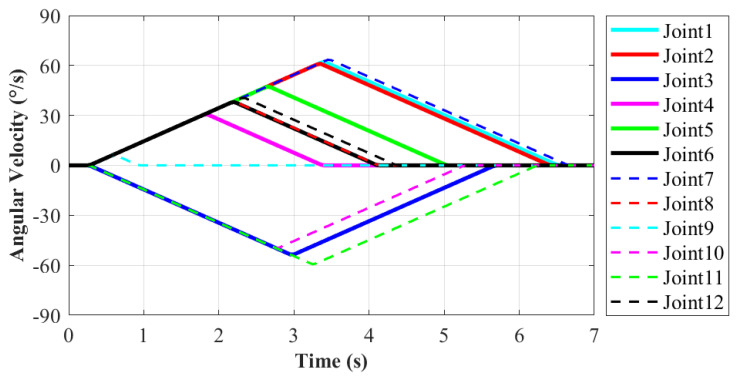
The angular velocity variation of each joint. (Action 9 according to Table 1).

**Figure 12:**
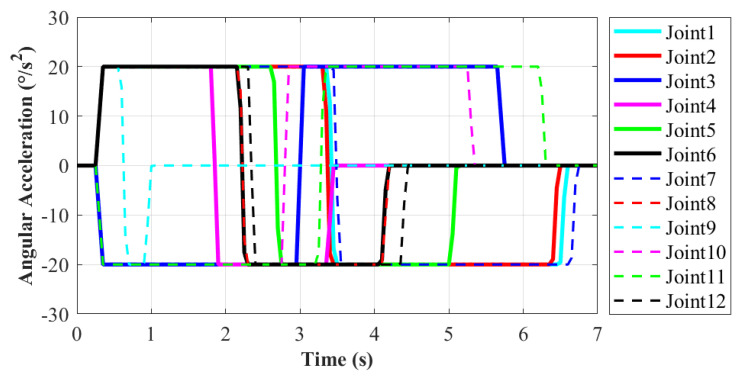
The angular acceleration variation of each joint. (Action 9 according to Table 1).

During the moving process, each joint movement of the robot has been recorded in order to evaluate the motion performance. The motion from State20 to State23 of the robot will be explained in detail, as shown in Figure 9e,i. In this process, the right end remains stationary, and the left end moves from work plane 2 to work plane 1. It enables the robot system to complete the conversion of work platform. The corresponding action is 11 according to Table 1. It is more difficult for the robot to move to a new work plane than working in the same one. Thus, this moving process is taken as an example to evaluate the motion performance of robot. The variations of each joint’s position, velocity and acceleration are shown in Figure 10, Figure 11 and Figure 12, respectively.

According to the motion planning, in order to make the motion relatively stable, the maximum angular velocity and acceleration of each joint are limited. The maximum angular velocity is limited to 90${}^{\circ }$/s, and the maximum angular acceleration is limited to 20${}^{\circ }$/s${}^{2}$. In the meanwhile, when in the starting or ending point, the angular velocity and acceleration of each joint are set to be zero. Thus, the robot joints will not produce excessive impact. According to the Figure 10, Figure 11 and Figure 12, the solid lines stand for Joint1–Joint6, which corresponds to the left limb. Similarly, the dashed lines represent Joint 7–Joint 12 of the right limb. The total moving time for this process is 6.5 s. The rotation range of each joint is −180–180${}^{\circ }$ based on the basic module. As shown in Figure 10, the minimum rotation angle is 2.1${}^{\circ }$ that occurs in Joint9. The maximum rotation angle is 203.6${}^{\circ }$ that belongs to Joint 7. It does not exceed the setting rotation range of the each joint, which can meet the requirements.

As shown in Figure 11, the minimum angular velocity is 6.3${}^{\circ }$/s at Joint9 and the maximum is 63.5${}^{\circ }$/s at Joint7, which also is consistent with the situation of joint angles. The curve of angular velocity is isosceles triangle, namely its motion process is divided into two stages with equal time. The velocity in the former stage increases uniformly to the maximum, and then it decreases uniformly to zero in the latter stage. The maximum velocity appears at the midpoint of the whole motion. In this way, it ensures the stability of the former and latter half of the motion and the robot approaches the target point slowly at the same time. According to Figure 12, the maximum angular acceleration of each joint is 20${}^{\circ }$/s${}^{2}$ as limited. Based on the variation demand of angular velocity, trapezoidal acceleration planning is adopted. When reaching the target point, the angular acceleration is reduced to zero to prevent collision with the handle on the work plane. In this paper, for the moving process on the ISS, the main goal is to ensure motion stability without pursuing excessively high velocity.The angular velocity and acceleration values of each joint are not set to be too large. In the future research, the robot motion will be further planned and optimized to fit actual application.

## 5. Conclusions

In this paper, a wearable robotic limbs system for astronaut has been proposed to assist the astronauts moving and working outside the space station cabin. For better service in the manned space field, the basic module units of robot are manufactured based on the modular design concept. Besides, the robot is trained to move autonomously by using reinforcement learning method. After the training, the acquisition skills of the robot are evaluated by setting the initial place randomly. For all the state points, the robot succeeds in moving autonomously from any starting place to the final target point. In the meanwhile, the basic moving actions of the robot are planned considering the motion stability and working comfort of the astronaut. In addition, a simulation platform of the robot and truss of ISS are built in the Vrep environment. The results of the reinforcement learning method and moving performance of the robot are verified in the simulation environment, which indicates the effectiveness of the reinforcement learning method for the robot to obtain the autonomous mobility towards the task target.

In future, the robot will be trained to adapt to the complex and changeable space environment. Combined with the prototype of the AstroLimbs, its motion assisting ability will be further verified in the low microgravity environment on the ground, which will provide ground test data for future application in outer space.

## Figures and Tables

**Table 1:** Determination of effective actions of the AstroLimbs.

No.	Eq-Coordinate	Action Mode	No.	Eq-Coordinate	Action Mode
1	{1, 0, 0}	*A*	8	{2, 1, −1}	*B*
2	{2, 0, 0}	*A*	9	{1, −1, 1}	*C*
3	{2, 1, 0}	*A*	10	{2, −1, 1}	*C*
4	{1, 1, 0}	*A*	11	{1, −1, −2}	*D*
5	{2, −1, 0}	*A*	12	{2, −1, −2}	*D*
6	{1, −1, 0}	*A*	13	{1, 1, 2}	*E*
7	{1, 1, −1}	*B*	14	{2, 1, 2}	*E*

## Data Availability

The data supporting reported results can be obtained in this article.
